# Evaluation of tolerability with the co-formulation elvitegravir, cobicistat, emtricitabine, and tenofovir disoproxil fumarate for post-HIV exposure prophylaxis

**DOI:** 10.1186/s12879-016-2056-3

**Published:** 2016-11-29

**Authors:** Nadia Valin, Laurent Fonquernie, Anne Daguenel, Pauline Campa, Theresita Anthony, Marguerite Guiguet, Pierre Marie Girard, Marie Caroline Meyohas

**Affiliations:** 1 Hôpital Saint-Antoine, Service des Maladies Infectieuses et Tropicales, 184 rue du Faubourg Saint-Antoine, F75571 Paris Cedex 12, France; 2 Hôpital Saint-Antoine, Service Pharmacie, 184 rue du Faubourg Saint-Antoine, F75571 Paris Cedex 12, France; 3 Sorbonne Universités, UPMC Univ Paris 06, INSERM, Institut Pierre Louis d’Epidemiologie et de Santé Publique (IPLESP UMRS 1136), F75013 Paris, France; 4 Sorbonne Universités, UPMC Univ Paris 06, F75005 Paris, France

## Abstract

**Background:**

The preferred regimen for HIV post-exposure prophylaxis (PEP) is based mainly on safety and tolerability because it is given to immunocompetent people without HIV infection for a limited time (28 days). The frequency of adverse events (AEs) may be > 60%. Although AEs are generally not severe, they can lead to lack of adherence and failure to complete the regimen. We evaluated the co-formulation elvitegravir, cobicistat, emtricitabine, and tenofovir disoproxil fumarate (Stribild^®^) prescribed as one pill taken once daily for HIV PEP in terms of tolerability and adherence.

**Methods:**

This was a prospective cohort study conducted in one hospital in Paris (April to December 2015. Each participant receiving the PEP treatment (FTC-150 mg/TDF-245 mg/elvitegravir-200 mg/cobicistat 150 mg once daily) at the pharmacy of the hospital were recruited consecutively. A clinical visit was planned at 8 weeks after sexual exposure. Reminders were sent to participants who missed the appointment. A standardized questionnaire was administered to evaluate completeness and tolerability at week 8.

**Results:**

Overall, 284 participants (86% men; 80% MSM; median age 30 years) were prescribed Stribild^®^; 50 stopped after reassessment of risk. Among 234 participants who effectively received PEP, 215 (92%) completed 28 days of PEP with only three who switched from Stribild^®^ to another PEP because of side effects. More than 60% of participants reported at least one AE, which were mild to moderate. Fatigue, central neurological and abdominal side effects were the most frequently reported.

**Conclusions:**

Stribild^®^ seems to be a good option for HIV PEP due to the easiness of use, the side effects profile and the high completion rate.

## Background

The preferred regimen for HIV post-exposure prophylaxis (PEP) has evolved recently, mainly due to the safety and tolerability of new antiretroviral drugs. PEP guidelines in the United States and Europe have been revised to recommend tenofovir disoproxil fumarate (TDF) and emtricitabine (FTC) as the preferred backbone drugs, with a protease inhibitor or an integrase inhibitor as the third drug [1–3], taking into account the short-term toxicity, cost, availability and possible risk of transmitted drug resistance in some contexts.

Antiretroviral drugs have well-documented toxicities and adverse events (AEs) in patients living with HIV/AIDS, but the occurrence of AEs during PEP might differ because the drugs are given to immunocompetent people without HIV infection for a limited time (28 days). The incidence of AEs related to PEP regimens may be > 60% with 3-drug regimens [4]. Although these AEs are generally not severe, experience suggests that they can lead to lack of adherence, and a substantial proportion of people for whom PEP is recommended fail to complete their prescribed regimen [5]. A simple and well-tolerated regimen may improve the acceptability and adherence of PEP.

In well-resourced settings, a recent policy shift for HIV PEP has been toward combining TDF and FTC with raltegravir as the third drug even if data are still limited in this indication [6]. The short-term tolerability of a regimen including an integrase inhibitor as a third agent has been reported in some studies of raltegravir [4, 7, 8]. The main side effects reported with this combination, are diarrhea (up to 55%), nausea (27%), headache (15%), fatigue (14%) and arthralgies and myalgies (8%). However, raltegravir is currently recommended to be prescribed twice daily, which may affect adherence. The association of TDF/FTC with elvitegravir and cobicistat, prescribed as one pill once daily could be more interesting for HIV PEP but is less well studied.

The current study evaluated this novel combined HIV PEP regimen in terms of tolerability and adherence.

## Methods

### Participants and setting

This prospective study included HIV-negative men and women ≥ 18 years old who were seen within 48 h after a potential sexual exposure to HIV-1; the sexual exposure could include anal, vaginal or oral sex, from an HIV-1-infected partner or high-risk partner of unknown HIV status [1].

Participants were consecutively recruited from St. Antoine Hospital in Paris between April 1, 2015 and December 31, 2015, in the emergency department during weekends, public holidays and evenings and in the infectious diseases department during the remaining time. PEP was systematically initiated when people presented in the emergency department and indications were re-assessed during a visit at Infectious Diseases department 5 days later at most. Thereafter, follow-up visits were scheduled for all participants in our HIV ambulatory care unit.

The study protocol was approved by the institutional review boards of St Antoine Hospital and the Pierre et Marie Curie University. All participants gave their consent to have their data recorded and analyzed anonymously.

### Study protocol

Trained physicians gave participants who were eligible for PEP treatment a standardized counseling message about potential signs and symptoms of AEs and acute HIV infection. Participants were given a printed card that indicated how to reach healthcare staff if necessary. Each participant received the PEP treatment (FTC-150 mg/TDF-245 mg/elvitegravir-200 mg/cobicistat 150 mg once daily) at the pharmacy of the hospital, where the 28-day treatment was delivered 3 times, on days 1, 8 and 15, and where the standardized questionnaire was administered by study staff to evaluate follow-up and tolerability of treatment during the month. Advice about medication uptake and side effects were also provided by pharmacists. A clinical visit was scheduled at week 8 after exposure, and blood testing was prescribed to examine biological variables, and hepatitis and HIV serology testing was prescribed on day 15 and week 8. In addition, participants who received the study drug were given a diary for recording AEs. Participants received a reminder call in case of a missed week-8 appointment (maximum 3 calls). Data about tolerability were self-reported by participants and were recorded during the pharmacy or clinical visit or by phone call. Adherence was determined by self-reporting the completion of the prophylaxis schedule. Data on AEs were graded according to the Division of AIDS Table for Grading the Severity of Adult and Paediatric Adverse Events and were classified as mild, moderate, severe and potentially life threatening (Version 1.0).

### Data analysis

Demographic, clinical, and behavioral data were collected in the Diamm^®^ (Micro 6, France) Database. If participants presented many times for sexual exposure risk, only the first episode was retained for analysis. All participants who received Stribild^®^ were included in the main analysis that take into account participants who were lost to follow-up with AE assumed (missing = failure). Results are presented as frequency and 95% confidence intervals (%). Fisher’s exact test was used to identify significant differences between categorical variables, and the Wilcoxon-Mann-Whitney test was used for continuous variables. Stata v14 was used to analyze data. *P* < 0.05 was considered statistically significant.

## Results

**Fig. 1:**
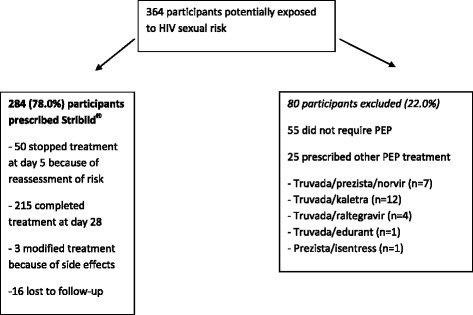
Flow chart of participants who consulted after sexual exposure to HIV infection in one Parisian hospital between April and December 2015. PEP: HIV post-exposure prophylaxis

Between April 1 and December 2015, a total of 364 potentially exposed participants were prescribed PEP (Fig. 1). Overall, 284 (78%) exposed participants received Stribild^®^ for 28 days; 50 participants stopped the PEP earlier because the sexual exposure was considered not at high risk. PEP was stopped earlier in people who had sex with people at low risk of transmission or with HIV patients with undetectable viral load since more than 6 months. These 284 participants were mostly men (86%), 80% of men who have sex with men and the median age was 30 years [range 18 to 69] without any difference between the participants who completed full course and participants who stopped earlier. A total of 48 patients (17%) indicated that they knew that they were exposed to an HIV-infected partner but this partner was not the regular partner for 34 cases (71%), with no data available on last viral load and current antiretroviral treatment. In total, 184 (65%) reported unprotected anal sex, 74 (26%) vaginal unprotected sex, 26 (9%) oral unprotected sex because of not using condoms or condom breakage; 142 (50%) reported known exposure to ejaculate. A total of 30 participants (11%) had a past sexual exposure to HIV with PEP. One participant had a positive hepatitis B antigen at the first visit.

Side effects could occur very early, and among the 50 participants who received < 5 days of PEP before stopping the treatment when the risk with sexual exposure was reassessed, 15 (30%; 95%CI, 17.9 to 44.6) reported experiencing at least one minor or moderate AE, mainly fatigue, nausea, or vomiting.

More than half of the 234 participants who were prescribed PEP for 28 days (*n* = 148, 63.2%; 95%CI, 56.7 to 69.4) reported at least one AEs. Among these participants, 3 stopped the drug after 2, 10 and 11 days because of moderate or severe side effects and were switched to another PEP treatment, and 16 participants were lost to follow-up after a median of 12 days. Data on tolerability were available for 218 (93%) participants: 100 participants provided tolerability information during the medical visit at week 8, 104 when visiting the pharmacy, and 14 participants provided information when contacted by phone call. Specific adverse events reported by participants are shown on Table 1. Fatigue and central neurological and abdominal side effects were the most frequent. Three participants showed minor liver cytolysis (<2.5 the upper limit of normal of AST or ALT). All these side effects were mild or moderate and tended to be self-limiting, not resulting in drug discontinuation except in 3 participants. These 3 participants had to switch their treatment because of moderate and severe side effects: 2 diarrhea and abdominal pain and 1 repeated vomiting episodes. AE occurrence did not differ by gender, age, type of exposure event or partner HIV status on statistical analysis. Overall, 11 participants went to their general practitioner for treatment of the side effects. No case of mortality due to AEs was reported, and all side effects were reversible at the end of treatment.

**Table 1:** Specific adverse events reported by participants who received a full course (28 days) or a short course (5 days) of the co-formulation elvitegravir, cobicistat, emtricitabine, and tenofovir disoproxil fumarate, one pill, once a day, as post-exposure prophylaxis (PEP) for sexual exposure to HIV infection

	28 days PEP	5 days PEP
Total	218 (100.0)^a^	50 (100.0)
At least one adverse event	132 (60.6 [54.0–67.1])	15 (30.0 [16.8–43.1])
Fatigue	57 (26.1 [20.3–32.0])	5 (10.0 [1.4–18.6])
Abdominal disorders		
- Nausea	55 (25.2 [19.4–31.0])	6 (12.0 [2.7–21.3])
- Vomiting	5 (2.3 [0.3–4.3])	4 (8.0 [0.2–15.8])
- Diarrhea	37 (17.0 [11.9–22.0])	1 (2.0 [0–6.0])
- Abdominal pain	18 (8.3 [4.6–11.9])	0
- Dysphagia	1 (0.5 [0–1.4])	0
- Dysgueusia	1 (0.5 [0–1.4])	0
Headache	17 (7.8 [4.2–11.4])	3 (6.0 [0–12.8])
Vertigo	5 (2.3 [0.3–4.3])	0
Insomnia	3 (1.4 [0–2.9])	0
Visual trouble	1 (0.5 [0–1.4])	0
Arthralgia or myalgia	2 (0.9 [0–2.2])	0
Fever sensation	3 (1.4 [0–2.9])	0
Rash	2 (0.9 [0–2.2])	0
Cytolysis	3 (1.4 [0–2.9])	0

Data are no. (%, 95% CI)

^a^Data on tolerability were missing for 16 participants who were prescribed 28 days of PEP and were lost to follow-up before the end of treatment

Among 215 participants who completed the PEP for 28 days, data on adherence were available for 195 (91%) and were considered complete or >95% for all. Results of HIV serology 2 months after the exposure were available for 131/234 participants (56%), and none had HIV infection. Among 50 participants who received < 5 days of the PEP before stopping when the risk had been reassessed, results of HIV serology 6 weeks after the exposure were available for 10 (20%), and none had HIV infection.

## Discussion

In this study of PEP with co-formulated elvitegravir, cobicistat, FTC and TDF prescribed as one pill once daily, side effects were reported by more than half of the participants, but nearly all completed the full 28 days of treatment with high adherence and only three had to switch because of side effects. Tolerability of this new regimen was unexpectedly no better than previously reported with various PEP regimens, but completion rate was higher. In seven PEP studies published since 2001, 51% of the total 1009 participants reported at least one AE and about 20% failed to complete the 28-day course [5, 9–14]. The most common side effects we found were similar to those reported in studies with raltegravir, with similar frequency of nausea, vomiting, diarrhea, abdominal discomfort, headache, and fatigue but less myalgias or arthralgias [6, 7], which were reported only twice in our study.

The 1% PEP discontinuation due to AEs we found was lower than the 20% discontinuation due to AEs with other standard regimens of ZDV + 3TC + LPV/r [5]. The discontinuation rate we observed was similar to that with raltegravir [7]. However, the simplicity of drug intake with only one pill daily could improve the treatment completion as compared with raltegravir, for which previous study reported approximately one quarter of patients consistently missing their second dose of raltegravir [7].

PEP based on the co-formulation of elvitegravir, cobicistat, FTC and TDF could be one option for PEP at least in some countries. Its availability is limited in resource-limited settings. In 2014 in France, the price for a 28 days full course of Stribild^®^ (980 euros) was close to the price of the previously recommanded association of Truvada^®^ and Prezista^®^/Norvir^®^ (1002 euros). Compared to the other single tablet regimen, Stribild^®^ was slightly more expensive than Eviplera^®^ (rilpivirine/TDF/FTC, 756 euros). However, this last association was not recommanded in case of high viral load (up to 100 000 copies per milliliters) which might occur in case of primoinfection and because of the higher risk of primary viral resistance of this non nucleosidic inhibitor. We need more head-to-head comparisons of completion rates and tolerability of PEP regimens, including newer integrase inhibitor-based regimens (ie, raltegravir vs dolutegravir vs elvitegravir) [15, 16].

French national guidelines for follow-up sexual exposure includes HIV antibody testing at baseline and 6 weeks or at baseline and 8 and 16 weeks after the exposure for people who did not or did receive PEP [1]. In our study, only 56% of people who were prescribed a complete PEP treatment underwent HIV-testing follow-up at 8 weeks and 20% of those who stopped PEP because of low risk. These low percentages are consistent with previous findings in PEP studies [17, 18]. Besides PEP prescription, good follow-up was previously found associated with older age and sexual encounter with a sex worker [19]. The completeness of HIV-testing follow-up could be increased by reducing the number of HIV testings with one HIV-serology testing 6 weeks after PEP completion, as proposed by the Swedish reference group for antiretroviral therapy [20], and targeting counseling to people receiving PEP.

Even if data on HIV testing follow-up are limited, the effectiveness of the co-formulation of elvitegravir, cobicistat, FTC and TDF as PEP appears to be good in our study since no HIV seroconversion occurred during the follow-up.

The main limitation of this study is the non comparative design of the study since all participants received the co-formulation of elvitegravir, cobicistat, FTC and TDF. Data on other comorbidities or concurrent medication which could have affected tolerability have not been collected. Another limitation is the low percentage of HIV serology follow-up which prevent us to obtain exhaustive data on Stribild^®^ effectiveness as post exposure prophylaxis.

## Conclusions

The co-formulation elvitegravir, cobicistat, FTC and TDF appeared to be a good option for HIV PEP in high-resource settings, with the need for appropriate and effective management of AEs, which remained frequent although mild.

## Abbreviations

AE: Adverse events
FTC: Emtricitabine
PEP: Post exposure prophylaxis
TDF: Tenofovir disoproxil fumarate
